# Genomic Evaluation of Assisted Gene Flow Options in an Endangered Rattlesnake

**DOI:** 10.1111/mec.70014

**Published:** 2025-07-07

**Authors:** Samarth Mathur, H. Lisle Gibbs

**Affiliations:** ^1^ Department of Evolution, Ecology, and Organismal Biology The Ohio State University Columbus Ohio USA; ^2^ Ohio Biodiversity Conservation Partnership The Ohio State University Columbus Ohio USA

**Keywords:** assisted gene flow, conservation genomics, functional genomic variation, genetic rescue, *Sistrurus catenatus*

## Abstract

Assisted gene flow is used to counteract genetic erosion in small populations of endangered species, yet an evaluation of genetic compatibility of potential donor populations and recipient populations is rare. We developed new metrics for assessing the genetic impact of genetic augmentation based on genotype identity of functional variants between donor and recipient genomes and used these metrics to evaluate options for assisted gene flow in Eastern Massasauga rattlesnake ( *Sistrurus catenatus* ) populations in Ohio, USA. We used putatively deleterious variants and genetic variants likely under positive selection (termed ‘adaptive’ variants) as the two components of functional variation. For potential donor and recipient populations, we estimated three key aspects of genetic compatibility: (a) introduction of novel variants, (b) masking or unmasking of existing deleterious variants and (c) potential for outbreeding depression through disruption of local adaptation. The main impact of augmentation from diverse donor populations was to introduce novel deleterious variants and to a lesser extent novel adaptive variants into each recipient population. Both donor populations had a similar minor impact in terms of masking existing deleterious variants. Finally, only ~7% of adaptive variants show evidence for local adaptation, arguing that the negative effect of outbreeding depression would be small. These results draw attention to the importance of considering the potential impact of both deleterious and adaptive genetic variants in augmentation efforts and suggest that in the case of these endangered rattlesnakes, the net effect of proposed assisted gene flow may lead to an increase in absolute levels of mutation load.

## 1 Introduction

Threatened and endangered species often exist in small populations that suffer from high levels of genetic erosion, which can negatively impact their future viability (Frankham et al. 2017; Leroy et al. 2017). Assisted gene flow is an in situ management strategy in which the genetic makeup of small recipient populations can be potentially improved by the introduction of individuals from larger and more genetically diverse populations. In theory, these introductions can mitigate the effects of high levels of genetic load and low levels of adaptive variation in recipient populations (Fitzpatrick et al. 2023; Frankham et al. 2011; Weeks et al. 2011) leading to the genetic rescue of recipient populations (Fitzpatrick et al. 2023; Whiteley et al. 2015). However, the positive effect of assisted gene flow is dependent on the genetic complementarity of donor and recipient populations in terms of whether the genetic input from donor individuals will minimise the negative impacts of genetic load while also increasing future viability by replenishing adaptive variation (Fitzpatrick and Funk 2021). Thus, evaluating the genetic compatibility of potential donor and recipient populations is a key step in identifying potentially successful genetic rescue options for a given species.

When considering appropriate strategies for assisted gene flow, it is important to evaluate the positive or negative genetic impacts that source populations will potentially have through the introduction of novel deleterious mutations and/or adaptive genetic variants (Fitzpatrick and Funk 2021). Positive impacts include the masking of recessive deleterious variants (in heterozygotes) that occur at high frequencies in the small recipient population and the introduction of adaptive variation previously lost due to genetic drift. However, these need to be balanced against potential risks, which include the introduction of new deleterious mutations leading to increased inbreeding depression and the introduction of variants that disrupt local adaptation, leading to outbreeding depression (Edmands 2017; Frankham et al. 2011). Until recently, the main options to identify suitable donor populations were to use population characteristics such as effective population size, levels of inbreeding, and gene flow, or use levels of neutral variation as a proxy for functional variation (Fitzpatrick and Funk 2021). However, these approaches lack information on the identity and abundance of the specific genetic variants that determine the levels of genetic erosion in each population. This makes it difficult to determine key aspects of the genetic impact of potential donor individuals, such as the number of novel mutations (positive or negative) that will be introduced, and whether the existing deleterious mutations being expressed in the recipient population due to inbreeding will be masked in heterozygotes. The recent availability of genomic information and methods for characterising positive and negative functional variation at a genome‐wide scale now makes it possible to indirectly assess the potential genetic impacts of specific donor populations on the functional genetic profile of recipient populations (e.g., Khan et al. 2021). These developments are timely as more species stand to benefit from a conservation strategy of assisted migration for genetic rescue than have previously been considered (Fitzpatrick et al. 2023).

Here, we apply a genomic‐based approach to evaluate assisted gene flow options for small populations by assessing allele‐specific impacts on genetic load and levels of adaptive variation. We identify putatively deleterious and adaptive variation in the genomes of multiple populations of the endangered Eastern Massasauga rattlesnake as previously described in Mathur et al. (2023) and use new metrics to assess the genomic compatibility between potential donor and recipient populations. This genomic compatibility is based on comparing the impacts of translocating individuals from specific donor populations to specific recipient populations in terms of the absolute number of new deleterious and adaptive variants introduced, the masking of existing deleterious mutations and the levels of local adaptation present in positively selected variation.

**FIGURE 1:**
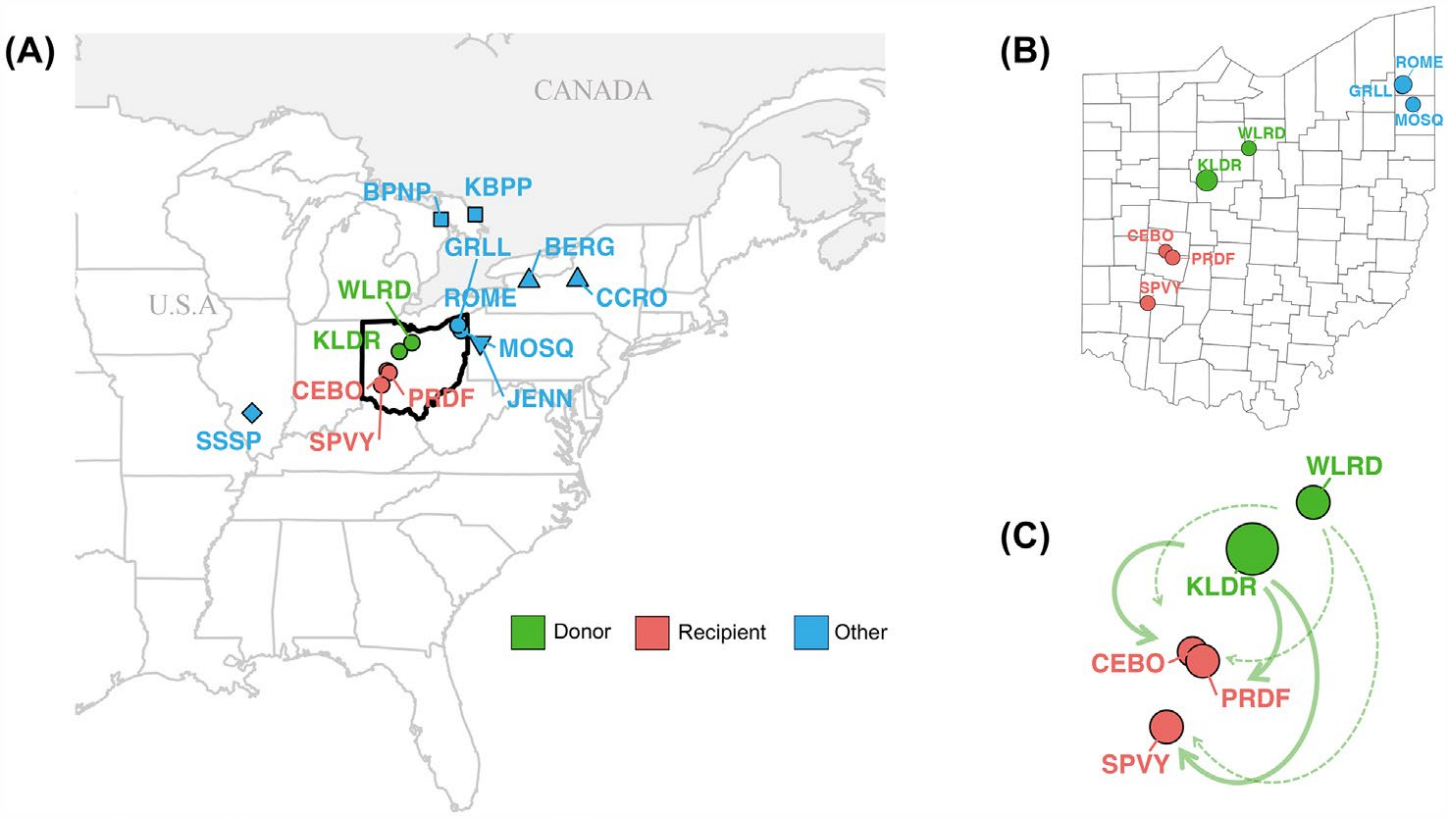
Sampling sites and potential donor and recipient sites for assisted gene flow of Eastern Massasauga rattlesnakes ( *Sistrurus catenatus* ) in the state of Ohio, USA. (A) One hundred and fifty‐two whole genomes were analysed to identify genome‐wide SNPs in different *S. catenatus* populations across the species range in the United States and Canada with (B) especially focusing on potential donor (green) and recipient (red) populations in the state of Ohio. (C) A schematic showing different assisted gene flow scenarios that are possible (see text). Population labels in red indicate the potential recipient populations, green are potential donor sites, and all other populations are labelled in blue.

The Eastern Massasauga Rattlesnake ( *Sistrurus catenatus* ) currently exists as a series of small, isolated populations across its range in the United States and Canada (Figure 1A). Population declines throughout its range due to habitat fragmentation and destruction have led to the listing of this species as Threatened under the United States Endangered Species Act (US Fish and Wildlife Service 2016) and as a Species at Risk in Canada (Government of Canada 2009). Recent work has shown that small populations of these snakes show high frequencies of deleterious mutations (Ochoa and Gibbs 2021) and reduced levels of adaptive variation (Ochoa et al. 2020). In Ohio, there are three populations which have exceptionally small recent effective population sizes (Mathur et al. 2023) and are potentially on the verge of extirpation (CEBO, SPVY and PRDF; Figure 1B). In contrast, there are several large populations which are sufficiently genetically diverse that they could serve as potential donor populations (KLDR and WLRD). The first steps in a conservation plan that includes assisted gene flow have been taken for one of these populations (G. Lipps, personal communication) yet no evaluation of the potential genetic consequences of this action is available. Our goal is to provide a genetic evaluation of various translocation scenarios that involve these populations (Figure 1C) which can then be considered along with other relevant political, ecological and logistic considerations for the implementation of the assisted gene flow efforts.

## 2 Materials and Methods

### 2.1 Samples, Sequencing and Sequence Processing

In this study, we analysed whole genome sequences from 152 *S. catenatus* individuals from 14 different study sites in the United States and Canada (Figure 1A; Table S1; Data S1). Our focus was on the isolated populations in Ohio (Figure 1B) to understand the consequences of assisted gene flow of moving snakes from larger and more genetically diverse (‘donor’) populations into the smaller and less diverse (‘recipient’) populations as part of a proposed assisted gene flow action within the state. We compared all possible donor–recipient scenarios (Figure 1C) of the two donor populations (KLDR and WLRD) and the three recipient populations (CEBO, PRDF and SPVY) to identify which donor population is more suitable and which recipient population would benefit the most from assisted gene flow.

We combined publicly available *S. catenatus* genome sequences (NCBI BioProject accession nos. PRJNA750087 and PRJNA975611) and new individuals sequenced for this study (Data S1). More details about DNA extraction and Illumina paired‐end (PE) 150‐bp reads sequencing are provided in Appendix S1. All sequences were mapped to the *S. catenatus* Hi‐C reference genome (GenBank accession no. GCA_037127405.1) using the germline pipeline available in NVIDIA Clara Parabricks v3.5, which is compatible with GATK4 best practices (Van der Auwera et al. 2018; Appendix S1). Details about sources of material for the reference genome are given in Mathur et al. (2023). The mean depth of coverage per individual was 15.3 ± 3.3× (mean ± SD) with 77.8% ± 9.6% of the reference genome covered at least 10× depth (Data S1).

### 2.2 Genotype Calling, Variant Filtration and Variant Annotation

We identified SNP variants using NVIDIA Clara Parabricks v3.5 germline pipeline and performed joint genotyping on all 152 samples using GATK v4.1.2.0 (McKenna et al. 2010; Appendix S1). We used ‘QD < 2.0; MQ < 20.0; |MQRankSum| > 3.0; |ReadPosRankSum| > 3.0; SOR > 5.0’ to filter low‐quality SNPs (Grossen et al. 2020). Only biallelic SNPs were retained and SNPs with genotypes missing in > 20% of samples, singletons and private doubletons were removed using VCFTools v0.1.16 (Danecek et al. 2011) resulting in the final set of 18,923,075 SNPs across the genome. We functionally annotated genome‐wide SNPs using SnpEff v.4.3 (Cingolani et al. 2014) and classified substitutions based on their regions (exons, introns and intergenic) and for exonic mutations, their functional class (missense, nonsense and silent). After filtering out low confidence annotations, we identified 68,041 missense, 1221 nonsense (hereafter referred to as ‘loss of function [LoF]’) and 93,195 silent substitutions in our dataset.

### 2.3 Identifying Functional Variants and Estimating Levels of Functional Genomic Variation

To identify missense mutations whose amino acid change can have a negative impact on overall protein function, we used the PROVEAN (Protein Variation Effect Analyzer) framework (de Brevern et al. 2012) as implemented in ePAT (extended PROVEAN annotation tool) software (Ito et al. 2021). PROVEAN predicts the impact of a functional or non‐silent mutation and amino acid substitution on the biological function of a protein by first aligning the protein sequences from related species using BLAST and then providing a quantitative measure (a PROVEAN score) of how phylogenetically conserved a missense allele is among homologous protein sequences. ePAT allows estimating PROVEAN scores for multiple variants simultaneously in a single step using batch processing. If the PROVEAN score is < −2.5, the missense mutation is considered as ‘damaging’ indicating a possibly negative impact of the amino acid change; whereas, missense mutations with a score > −2.5 are considered ‘neutral’ with no effect of the amino acid change on the protein function (Ito et al. 2021). Out of 68,041 missense mutations within our dataset, only 6623 mutations (~10%) were classified as damaging. We considered both damaging missense and LoF substitutions as deleterious mutations because both these types of substitutions can negatively impact individual fitness (through possible sublethal and lethal effects, respectively) and thus contribute to the overall mutation load. We estimated the mutation load of deleterious mutations at an individual level by partitioning the two components by the zygosity of deleterious alleles: Masked load (*L*_mask_) and realised load (*L*_realised_). For each individual genome, *L*_mask_ is the total number of deleterious SNPs that are present as heterozygotes, whereas *L*_realised_ is the total number of SNPs that are homozygous for the deleterious allele (Bertorelle et al. 2022; Mathur and DeWoody 2021). We used the mean individual load as an estimate of population levels of mutation load.

To identify putative adaptive variation, we first identified non‐synonymous substitutions in protein‐coding regions of genes previously determined to be under positive selection in *S. catenatus* based on the Direction of Selection (‘DoS’) values (Stoletzki and Eyre‐Walker 2010; see Mathur et al. 2023 for details; also see Appendix S1). We refer to these non‐synonymous substitutions as positively selected variants to better represent the uncertainty about whether they do in fact represent adaptive variation (see Mathur et al. 2023 for discussion).

### 2.4 Assessing the Genomic Compatibility Between Donor and Recipient Populations

Mean levels of mutational load and positively selected variation within each population fail to account for the genotype identity of these substitutions between a pair of genomes and their overall abundance in donor and recipient populations. This is important because evaluating the impact of a potential donor on a recipient population includes identifying whether novel mutations (positive or negative) will be introduced and if a deleterious mutation that exists as homozygote (i.e., realised) in a recipient individual will be masked as heterozygote in the next (F1) generation (Khan et al. 2021). Genomic information provides this added nuance by comparing genotypic differences in various donor and recipient genomes at positive and negative functional loci.

**FIGURE 2:**
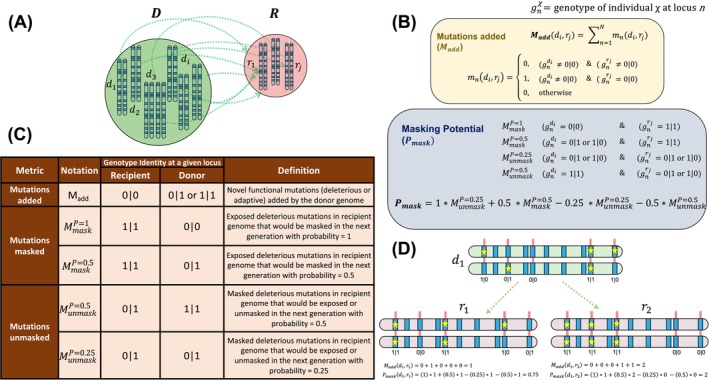
Estimating genomic compatibility for assisted gene flow between a donor and a recipient population. (A) Genomic compatibility is defined between a Donor (D) and Recipient (R) population by comparing the functional genotypes between each pair of donor (*d*_1_, *d*_2_, …, *d*_*i*_) and recipient (*r*_1_, *r*_2_, …, *r*_*j*_) genomes (Table S1). (B) To estimate genomic compatibility, we defined two metrics: Mutations added (*M*_add_) that measures the number of unique deleterious or adaptive alleles present in a donor genome that can be added to the recipient genome, and masking potential (*P*_mask_) that estimates a cumulative score of the probabilities of masking a homozygous allele (*P*_mask_ > 0) and unmasking a heterozygote allele (*P*_mask_ < 0). (C) Table describing the metrics to count different category of mutations between a donor and recipient genome based on the genotype identity of the functional mutation. See Appendix S1 for more details. (D) An example illustrating how the two genetic compatibility metrics can be estimated between a donor (*d*_1_) and two different recipient (*r*_1_ and *r*_2_) genomes by comparing the genotype combinations of derived allele (star) at different loci of interest.

For this purpose, we developed two new indices that compare the genomic compatibility between a pair of donor and recipient populations based on the genotypes of different functional mutations as outlined in Figure 2: (a) mutations added (*M*_add_), the number of novel negative or positive mutations added by a donor to the recipient population, and (b) masking potential (*P*_mask_), the net effect of the number of exposed negative variants in a recipient population that can be masked by individuals from the donor population and the number of already masked negative variants in recipient individuals that can be unmasked or exposed by the donor individual in the next generation (Figure 2B). Exposed mutations can be masked at different probabilities (*p*) in the F1 generation depending on donor genotypes (*p* = 1 or *p* = 0.5) and similarly, masked mutations can be exposed at different probabilities as well (*p* = 0.5, *p* = 0.25; Figure 2C), assuming Mendelian inheritance, that is, for all loci, the two alleles would segregate in equal proportions in the gametes and all genotype combinations in the F1 generation can theoretically exist. *P*_mask_ estimates a cumulative score of the probabilities of a donor genome masking homozygous alleles (*P*_mask_ > 0) and unmasking heterozygote alleles (*P*_mask_ < 0) when crossed with a recipient genome, considering the probability weights of masked and unmasked mutations. We then applied these metrics to evaluate the potential consequences of assisted gene flow for the different donor–recipient scenarios as described in Figure 1C. With the introduction of masking potential (*P*_mask_), we assume that masking of deleterious alleles in heterozygotes would result in higher fitness as compared to exposed deleterious alleles in homozygous mutant genotypes (see Appendix S1 for more details). Moreover, we emphasise that our approach is computational and the overall fitness impact of masking/unmasking deleterious alleles on an individual would depend on the empirical strength of selection of each the positive and negative variants that we analysed in our study.

### 2.5 Evidence for Local Adaptation Driving Genetic Differentiation Between Ohio Populations

The introduction of a novel adaptive allele to a recipient population is generally assumed to have a positive impact on future population viability (Khan et al. 2021). If local adaptation is high among populations, the introduction of novel adaptive alleles could potentially have a negative impact through the disruption of locally adapted gene complexes or the introduction of maladapted alleles leading to outbreeding depression (Brooks et al. 2024; Edmands 2007). Identifying local adaptation as a selective pressure leading to adaptive differences between isolated populations is challenging (Hoban et al. 2016). We can assess the possible role of local adaptation as a driver of population differences by comparing the differentiation in positive variants against background differentiation in neutral loci (which reflect non‐adaptive processes alone). Functional variants that are positively selected in *S. catenatus* and also show greater levels of divergence than that observed at neutral loci are candidates for functional loci that may also have contributed to local adaptation in different populations. The relative abundance of such variants represents an estimate of the importance of local adaptation as an evolutionary process in these populations (Margres et al. 2017).

We performed an outlier test by estimating the *F*_ST_ value of each adaptive locus among all Ohio populations and comparing it to the *F*_ST_ value of loci in gene desert regions (i.e., regions that lie in intergenic regions outside of any possible physical linkage to any protein‐coding regions; Appendix S1). Loci evolving via local adaptation among these populations were identified as having *F*_ST_ values that were greater than the 95th percentile of the neutral locus *F*_ST_ distribution (Margres et al. 2017; Ochoa et al. 2020).

## 3 Results

### 3.1 Levels of Functional Diversity

We first compared population level estimates of negative and positive functional diversity among all *S. catenatus* populations to get a baseline estimate of different components of genomic diversity. We determined that differences in genetic load are present among different Ohio populations (Figure S1; Table S2). Mean population levels of realised genetic load for both high impact (LoF; *N* = 1221) and lower impact (damaging missense based on PROVEAN scores; *N* = 6623) deleterious mutations are lower in the two potential donor populations compared to each of the three recipient populations (Figure S1) and snakes from at least one potential recipient population (CEBO) had high mean realised load (Table S2). In contrast, mean levels of adaptive variation are similar among the different populations (Table S2). The KLDR donor population shows slightly higher mean levels of diversity in positive variants than mean values for any of the recipient populations. In contrast, the WLRD population has a level of diversity in positive variants similar to (CEBO) or less than (PRDF and SPVY) the mean values for the potential recipient populations (Table S2). Based on these results, KLDR individuals are predicted to provide an advantage over WLRD individuals in terms of potentially increasing adaptive diversity in recipient populations. However, all these comparisons of both negative and positive genetic variants fail to consider the impact of the identity of specific genetic variants in donor and recipient populations.

### 3.2 Introduction of Novel Mutations

**FIGURE 3:**
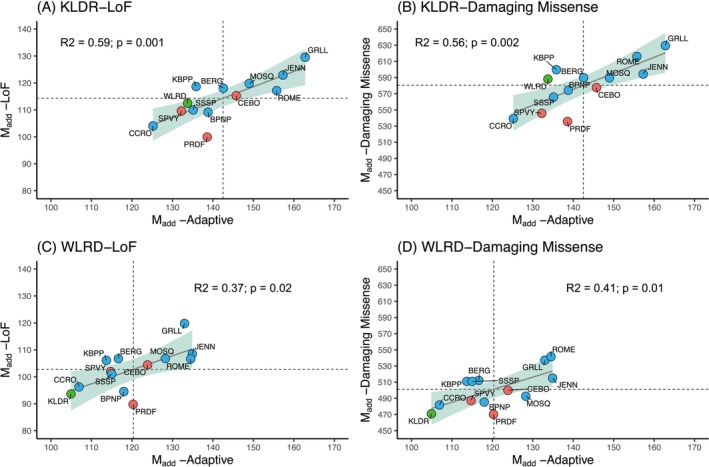
Genetic compatibility assessment based on novel adaptive variants and deleterious mutations added by donor to target recipient (and other) populations. Mean number of novel adaptive variants (*x*‐axis) versus (A) LoF and (B) damaging missense mutations (*y*‐axis) introduced by KLDR genomes that are absent in *S. catenatus* genomes from other populations (*M*_add_). (C, D) Similar analysis was performed using WLRD genomes as donors.

Next, we computationally assessed the effect of assisted gene flow involving moving snakes from donor (KLDR and WLRD) to recipient populations (CEBO, PRDF and SPVY) using the different genetic compatibility metrics described in Figure 2. First, we estimated the assisted gene flow impact in terms of introducing novel functional variants (*M*_add_), both deleterious and positive. Our analysis shows that introducing individuals from either donor population would add novel positive and negative functional variants, which can impact the genetic fitness of the recipient populations (Table S3) and that the number of each type of mutation introduced is positively correlated (Figure 3). A significant positive relationship between the number of novel positive mutations (*M*_add‐Adaptive_) and deleterious mutations (both LoF *M*_add‐LoF_ and damaging missense *M*_add‐damaging_) added by donor genomes means that the donor population that would add more novel adaptive variants into the recipient population would also lead to the introduction of more new deleterious mutations (Figure 3). However, in terms of absolute numbers, most of the novel variants added (~80%) will be deleterious mutations in the form of damaging missense variants (Figure 3B,D) with a smaller number of LoF mutations also added (Figure 3A,C; Table S3). Thus, assisted gene flow would have both negative and positive impacts, but in terms of the numbers of negative variants added, the number of low‐impact variants introduced would be about fivefold higher than the number of high‐impact deleterious mutations.

In terms of relative impacts of the two donors, an average KLDR snake would add more new LoF mutations (100–115) into the three recipient populations relative to an average WLDR individual (90–104 new mutations) and more lower impact missense mutations (536–578) relative to a WLRD individual (470–500 new mutations; Table S2). Relative to total functional mutations already present in recipient genomes, using KLDR snakes as donors would result in 28%–34% more novel LoF and 34%–39% damaging missense mutations being introduced to the recipient populations whereas WLRD would add 26%–32% novel LoF and 31%–36% damaging missense mutations (Table S4). In contrast, KLDR individuals will add more positive variants (132–146; 32%–36%) to recipient populations than would an average WLDR individual (115–124; 29%–33%) although the difference is small. Overall, these results indicate that the impacts on functional genomic variation in donor populations of choosing one donor population over the other are small in terms of adding novel mutations.

### 3.3 Masking and Unmasking of Deleterious Mutations, and Overall Masking Potential

Overall masking potential (*P*_mask_) of a donor population is dependent on how many deleterious alleles that are exposed in homozygotes would get masked in heterozygotes and how many deleterious alleles that are already masked would get exposed in the next generation (F1 generation). This metric accounts for the differences in masking/unmasking probabilities between the genotype identity of each locus between the donor and recipient genome (Figure 2; Appendix S1). Figure S2 and Table S5 report the number of deleterious mutations that are potentially masked (at *p* = 1 and 0.5; see Figure 2C) and unmasked (at *p* = 0.5 and 0.25; Figure 2C) for both LoF and missense mutations. Overall, when considering the mutation load (masked and realised) which is already present in each individual recipient genome, 27%–34% of the exposed LoF and 34%–42% of damaging missense mutations will likely be masked (at *p* = 1) by KLDR genomes (Table S6). Similarly, only 14.1%–14.3% of the masked LoF and 7.4%–8.1% damaging missense mutations are likely to get exposed (at *p* = 0.5) by introducing KLDR genomes. Proportions were similar but slightly lower for WLRD donors (Table S6).

We used *P*_mask_ to summarise the net potential of an average donor genome to mask or unmask deleterious mutations across the different probabilities described in Figure 2C (Figure S3; Table S7). The results show that the degree of masking varies markedly across potential recipient populations but is similar between potential donor populations. For LoF mutations, the degree of masking is highest for the CEBO, intermediate for SPVY and lowest for the PRDF recipient population. There was a similar pattern for damaging missense mutations.

### 3.4 Net Impact of Assisted Gene Flow

**FIGURE 4:**
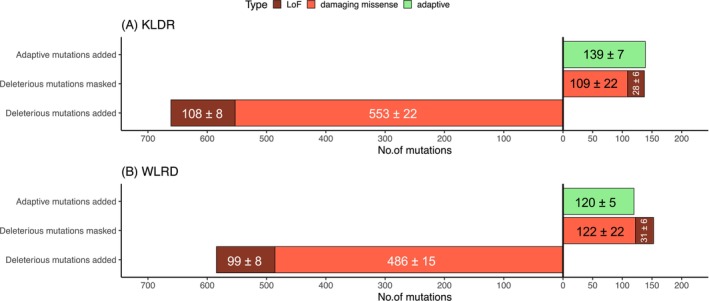
Summary of impact of assisted gene flow on recipient populations in terms of numbers of variants with negative and positive fitness‐related effects. The bar plots represent the mean number of variants added or masked (at *p* = 1) averaged over the three recipient populations when augmented with (A) KLDR or (B) WLRD donor population. Note that the estimate of numbers of deleterious mutations masked is a minimum estimate because only mutations with masking probability of *p* = 1. Numbers indicate mean ± 1 SD.

Figure 4 summarises the net impact of potential assisted gene flow by KLDR and WLDR individuals across recipient populations in terms of changes in numbers of negative and positive genetic variants. When used as a donor, an average individual from KLDR will introduce 139 ± 7 novel positive variants and 553 ± 22 novel damaging missense and 108 ± 8 LoF variants. Additionally, when the degree of masking is considered (*p* = 1), KLDR genomes will mask pre‐existing 28 ± 6 LoF and 109 ± 22 damaging missense mutations that are exposed in the recipient populations. Values for donor individuals from WLRD are smaller but similar in relative magnitude: 120 ± 5 new positive variants, 486 ± 15 damaging missense and 99 ± 8 LoF mutations would be introduced; 31 ± 6 pre‐existing LoF and 122 ± 22 damaging missense mutations that are exposed would be masked. Overall, our mutation counting approach indicates that the introduction of donor individuals from both source populations results in a > 4.5‐fold increase in the relative number of negative (high and low impact) to positive genetic mutations in potential donor populations. Most of these variants are low‐impact missense mutations. When only high‐impact LoF negative mutations are considered, the numbers of negative versus positive mutations are similar, with slightly more positive variants being added.

### 3.5 Impact of Local Adaptation

**FIGURE 5:**
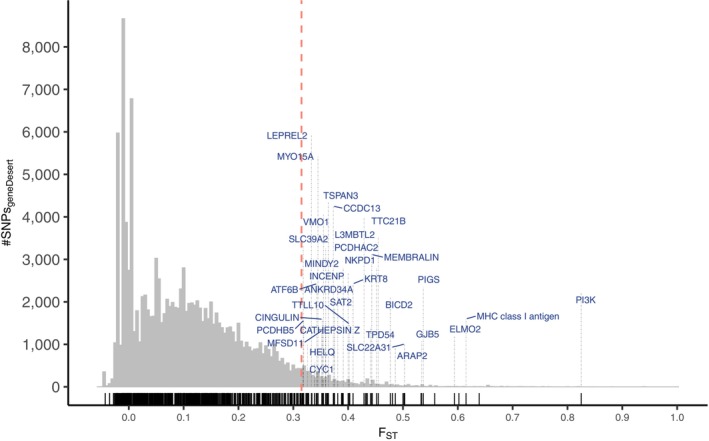
Population outlier analysis based on *F*_ST_ frequency histograms of functional and neutral sites in *S. catenatus* populations in Ohio. Light grey bars represent *F*_ST_ distribution of SNPs in gene desert regions of the genome; red dashed lines indicate the 95th percentile *F*_ST_ value in gene desert regions. Black vertical lines at the bottom of each plot denote *F*_ST_ estimates at each missense SNP in the *S. catenatus* adaptive genes (Mathur et al. 2023). Adaptive genes that contain SNPs with *F*_ST_ > 95th percentile of gene desert SNPs are labelled in blue. The per‐SNP *F*_ST_ for each SNP was calculated only using OH samples (SNP_geneDesert_ = 123,754; SNP_adaptive_ = 1053; SNP_adaptive_ in top 5% of the gene desert *F*_ST_ distribution = 78).

Our outlier analysis suggests that relatively few variants putatively under positive selection in *S. catenatus* show evidence for local adaptation among Ohio populations, and so possible negative effects due to outbreeding depression are likely small. Figure 5 shows distributions of *F*_ST_ values for neutral variants from ‘gene desert’ non‐coding regions and for missense variants in coding regions of genes under strong positive selection (Mathur et al. 2023) for Ohio populations. Only a small number of positive variants show elevated *F*_ST_ values consistent with local adaptation: specifically, only 78 of 1053 total positive variants (7.4%) showed *F*_ST_ values greater than the 95% cut‐off based on the null distribution for neutral variants in these populations. These results suggest that local adaptation may only be responsible for differentiation in ~7% of the putative positive variants in our study, and so the potential for outbreeding depression because of assisted gene flow is small.

## 4 Discussion

### 4.1 Potential Genetic Impacts of Assisted Gene Flow

Traditionally, the potential genetic impacts of population translocations into small, inbred populations of endangered species have relied on indirect measures of key population genetic parameters (Fitzpatrick and Funk 2021). Evaluation of specific assisted gene flow options using genomic information enables more direct analyses of genetic consequences of these options in terms of specific ‘good’ and ‘bad’ functional variants (Fitzpatrick and Funk 2021; Hedrick et al. 2013; Khan et al. 2021; Quinn et al. 2024). This leads to quantitative estimates of the relative magnitude of specific genomic contributions that potentially affect short‐ and long‐term population survival.

Our newly developed genomic compatibility approach (Figure 2) indicates that the net effect of proposed assisted gene flow options for Eastern Massasauga rattlesnake populations in Ohio is: (a) a large increase in the absolute number of novel deleterious mutations introduced into recipient populations, (b) a relatively smaller increase in the number of novel putatively adaptive variants introduced and (c) minor impacts of the masking or unmasking of deleterious alleles in the first generation post‐augmentation.

The impacts on the levels of functional genetic variation in the recipient genomes would be substantial. Based on the average number of variants present genome wide in a recipient individual (positive = 274 ± 38, damaging missense = 978 ± 133, LoF = 234 ± 28), KLDR donors will lead to a 33.7% ± 2.3% increase in the numbers of positive variants, a 36.2% ± 2.5% increase in damaging missense mutations and a 31.6% ± 2.9% increase in LoF mutations in recipient individuals (Figure 4). However, the absolute number of mutations present is more relevant to genome‐wide fitness effects and a > 4.5‐fold increase in the absolute number of negative versus positive mutations in recipient populations suggests that, based on absolute numbers alone, the net impact of augmentation would be to increase the mutation load in recipient populations. These insights would not be possible without a locus‐by‐locus assessment of the genotypic identity of functional variants and their possible fate upon augmentation.

Currently, we have no way to evaluate the negative and positive impacts of specific mutations on survival and reproduction in these snakes, and so our inferences are based on numbers of mutations alone. Yet fitness differences between mutations likely exist, as incorporated in empirical distributions of fitness effects for model organisms (Eyre‐Walker and Keightley 2007). As such, we cannot exclude the possibility that the average fitness effect of an adaptive variant is higher than the negative fitness effect of a deleterious mutations. This could lead to the situation whereby assisted gene flow may lead to a net fitness benefit to recipient populations, despite the lower number of adaptive variants introduced relative to the number of deleterious variants. Many empirical studies of assisted gene flow to date show an increase in the viability of recipient populations (Clarke et al. 2024; Ralls et al. 2020) which is consistent with a broad positive impact of this management action in small populations through either the addition of new adaptive variants or the masking of existing deleterious mutations in heterozygotes. It is also consistent with an overwhelming benefit of demographic effects alone, through the increased buffering of small recipient populations against stochastic events and allele effects due to increase in population sizes (Hufbauer et al. 2015).

For negative mutations, we attempted to add context by classifying variants into high impact (LoF) and lower impact (damaging missense) mutations, but these classifications are not linked to demographic data, which is difficult to obtain in these cryptic species. An alternative for assessing the fitness effect of deleterious alleles would be to use an approach such as the Combined Annotation‐Dependent Depletion (CADD) framework (Rentzsch et al. 2019; see for example Speak et al. 2024) but this requires information on the phenotypic impact of specific mutations for a related model organism, which does not currently exist for a reptile species.

Estimating plausible positive or negative fitness effects for substitutions that are computationally inferred is a general problem in using genomic‐based approaches for assessing functional variation (Galtier and Duret 2007; Kardos et al. 2024; Orr 2010; Robinson et al. 2023). As discussed in Mathur et al. (2023), the impact of a given missense mutation considered in this study could be influenced by a number of factors. These include compensatory mutations which could modify the effect of substitutions our scheme classifies and factors such as dominance, epistasis, pleiotropy and purging may also complicate the relationship between mutation load, adaptive diversity and fitness (Grossen et al. 2020). Geographic variation in habitat can also alter the impact of deleterious or adaptive mutations among populations (Mee and Yeaman 2019) although the impacts of local adaption on our positive variants seem limited (Figure 5). Finally, it also seems unlikely that our approach detects all adaptive variants that could arise under different types of selection such as frequency dependent selection because we have focused on measuring variants that increase levels of functional polymorphism in populations (Mathur et al. 2023).

Our quantitative approach addresses a long‐standing question about the relative importance of the introduction of novel deleterious alleles compared to the masking of pre‐existing deleterious variants (Fitzpatrick and Funk 2021; Khan et al. 2021). Our results demonstrate that, for this species, masking also represents a moderate advantage of assisted gene flow resulting in the neutralising of 26%–36% of LoF mutations and 34%–46% of damaging missense variants (Table S6) present in small populations of these snakes. Based on numbers alone, the introduction of novel deleterious alleles has a greater impact on the genetic makeup of small populations and so should be of greater focus when designing and assessing assisted gene flow plans for this species.

Finally, our analyses suggest that the potential for outbreeding depression is low in this set of populations because relatively few of the positively selected variants in *S. catenatus* that we have analysed show evidence for local adaptation in Ohio populations (Figure 5) at least over the limited spatial scale that we conducted these analyses. This is significant because reptiles in general show limited dispersal, which can lead to fine‐scale local adaptation (Brooks et al. 2024). More generally, a commonly cited reason for not pursuing assisted gene flow in endangered species is the fear of disrupting local adaptation in recipient populations (Bell et al. 2019; Ralls et al. 2020). Our analyses used methods for assessing local adaptation over evolutionary timescales, and our results suggest that concern about outbreeding depression in these snakes is likely minimal.

We recognise that detecting local adaptation using *F*_ST_ outlier tests has significant limitations, and the results presented here need to be viewed as tentative and need to be confirmed with additional analyses (Hoban et al. 2016). In particular, outlier tests can be prone to false positives due to the influence of demographic history, background selection and neutral evolutionary processes that can falsely mimic the effects of selection and fail to account for scenarios where selection favours different alleles in different populations or when the genetic architecture of local adaptation involves many loci of small effect (Hoban et al. 2016; Whitlock and Lotterhos 2015). Future approaches could involve using more refined null models that incorporate more detailed genomic and demographic information specific to this species (Johri et al. 2022) and conducting gene–environment association studies to link variation in specific loci with environmental variation to build the case that local environmental variation underlies selection for genic differentiation between populations (Hoban et al. 2016).

Our results add to the increasing recognition among conservation biologists that using donors from large genetically diverse populations can have both positive and negative genetic impacts on recipient populations (Hedrick et al. 2013; Khan et al. 2021). A long‐held view is that large genetically diverse populations are good source populations for assisted gene flow because of the increase in diversity that will occur in small, depleted populations (e.g., Fitzpatrick and Funk 2021). This reasoning makes the implicit assumption that adaptive variation will be the primary form of functional variation that will be transferred. However, large genetically diverse populations also contain high levels of potential inbreeding load in the form of deleterious variants hidden in heterozygous individuals (Mathur and DeWoody 2021) that are then exposed in small populations through interbreeding with inbred recipient individuals (Kardos et al. 2021; Mathur et al. 2023). We suspect that these hidden variants are the primary source of the new deleterious variants that will be transferred from donor to recipient populations in these snakes.

Recognising that large genetically diverse populations contain higher levels of both ‘good’ and ‘bad’ variation is important when assessing assisted gene flow options in other endangered species. Our genomic compatibility approach allows a quantitative assessment of the relative impacts of both components of functional variation between donor and recipient populations and indicates that, at least in this species with this genetic architecture, substantially more deleterious mutations would be introduced than adaptive variants. Future extensions of this work could include using individual‐based simulations with estimates of Distribution of Fitness Effects (DFE) to explore how the net impact of positive and negative mutations in donor individuals varies under different demographic scenarios (e.g., Kyriazis et al. 2024) and genetic backgrounds in recipient populations.

### 4.2 Implications for Assisted Gene Flow in Snake Populations

Our results suggest that the net effect of proposed assisted gene flow for the three small Ohio populations of *S. catenatus* will be an increase in the total number of mostly small effect deleterious mutations and a smaller increase in the number of presumed adaptive variants. Based on variant counts alone, this suggests that in this specific situation augmentation will have a negative impact on the genetic diversity present in these small populations. However, this conclusion is qualified by the fact that there is a lack of difficult‐to‐obtain phenotypic data that is necessary to evaluate the fitness impacts of specific functional mutations. These impacts are similar between the two potential donor populations analysed, indicating that from a purely genetic perspective there is little basis for choosing individuals from one population over the other for use as donors. We also acknowledge that genetic considerations are only one aspect in the choice of whether assisted gene flow should be pursued in a given situation, and ecological, political and logistic considerations are also important components of when deciding the most suitable donor population(s) (Seddon et al. 2014). Finally, the complete isolation between many existing *S. catenatus* populations throughout much of their range means that translocations may be the only way in which specific populations may survive through the positive impacts of demographic rescue, even in the face of possible negative genetic impacts. This emphasises the need to evaluate all potential risks and benefits—genetic and demographic—using an appropriate risk‐management framework (Liddell et al. 2021) when making decisions about whether population augmentation should be pursued for a given species, including Eastern Massasauga rattlesnakes.

## Author Contributions

S.M. and H.L.G. designed the research; S.M. performed the research and analysed the data; S.M. and H.L.G. wrote the paper.

## Conflicts of Interest

The authors declare no conflicts of interest.

## Supporting information


Appendix S1

Appendix S2

## Data Availability

Raw sequence data used in this study are available in NCBI's Short Read Archive BioProject accession no. PRJNA1220313. The scripts developed for data analysis and visualisation and additional data can be publicly accessed on GitHub at https://github.com/samarth8392/genomic_compatibility.
