# Supplementary material for: Genomic Evaluation of Assisted Gene Flow Options in an Endangered Rattlesnake
Source: Mol Ecol. 2025 Jul 7;34(16):e70014. doi: 10.1111/mec.70014 (PMC12329626; doi:10.1111/mec.70014)
Supplement: Supplementary file 1 — Appendix S1 [file MEC-34-e70014-s001.pdf]

Supplementary Information

Genomic evaluation of assisted gene flow options in an endangered rattlesnake.

Samarth Mathur[1,2], and H. Lisle Gibbs[1,2*]

[1]*Department of Evolution, Ecology, and Organismal Biology, The Ohio State University, Columbus, OH, USA*

[2]*Ohio Biodiversity Conservation Partnership, The Ohio State University, Columbus, OH, USA*

* Corresponding author: H. Lisle Gibbs.

Email: gibbs.128@osu.edu

Supporting Information Text

DNA Extraction and library preparation for genome sequencing.

Approximately 50 µL of whole blood was mixed with 700 µL lysis buffer and 20 µL of 20 ng/µL of Proteinase K then incubated for approximately three hours. After digestion, 700 µL of Phenol were added, the solution of mixed vigorously, and then centrifuged at 13,000 rpm for five minutes. The aqueous layer containing the DNA was removed, 700 µL of Phenol:Chloroform:Isoamyl were added, and mixing and centrifuging was repeated. This procedure was repeated twice more adding 700 µL of Chloroform:Isoamyl after which 600 µL of DNA containing supernatant were removed. We then added 50 µL of 3M sodium acetate and 1000 µL of ice-cold ethanol to precipitate DNA. The DNA pellet was washed with 70% ethanol and then resuspended in Tris-HCl pH=8.

The Illumina 150bp paired-end shotgun genomic libraries were prepared with the Hyper Library construction kit from Kapa Biosystems (Roche). The libraries were pooled; quantitated by qPCR and sequenced on one S4 lane for 151 cycles from both ends of the fragments on a NovaSeq 6000. Fastq files were generated and demultiplexed with with the bcl2fastq v2.20 Conversion Software (Illumina).

Identifying putative adaptive variants.

To identify SNP variants that plausibly represent adaptive variation we followed the logic and protocol described in detail by Mathur et al. (2023). Briefly, we assumed that nonsynonymous variants in genes that were under strong positive selection during divergence between sister taxa also represent adaptive mutations within each population. This assumption is based on the expectation that the evolutionary history of selection on a gene at the interspecific level will also reflect the type of selection acting at the intraspecific level as recently demonstrated for some mammals (Latrille, Rodrigue, & Lartillot, 2023). We estimated the type and strength of selection acting on all annotated protein coding genes by calculating the modified Neutrality Index called Direction of Selection (DoS; (Stoletzki & Eyre-Walker, 2010)) using polymorphism and divergence data from *S. catenatus* and *S. tergeminus*. For a gene, DoS can be estimated as:

$$DoS = \frac{D_n}{D_n + D_s} - \frac{P_n}{P_n + P_s}$$

where D_n is the number of nonsynonymous substitutions between *S. catenatus* and *S. tergeminus*, D_s is the number of synonymous substitutions between *S. catenatus* and *S. tergeminus* within the gene, P_n is the number of nonsynonymous polymorphisms within *S. catenatus*, and P_s is the number of synonymous polymorphisms within *S. catenatus*. To minimize drift effects on intraspecific polymorphism data we used polymorphism data from the KLDR population of *S. catenatus* which has a relatively large effective population size and a reference population of the outbred sister species, *S. tergeminus* from Cheyenne Bottoms to estimate divergence (for details see Mathur et al. 2023). We removed genes with undefined DoS and further restricted our analyses to genes with $P_n + D_n \geq 4$ which are potentially informative about selection. Finally, we designated nonsynonymous substitutions in protein coding regions of the genes with highest 10% of DoS values as possible adaptive variants.

Assessing the genomic compatibility between donor and recipient populations for genetic augmentation.

We assessed genetic compatibility of donor and recipient populations using the following metrics based on the identify of individual mutations (summarized in Figure 2).

Mutations added (M_{add}). Conceptually, M_{add} measures the number of loci where the donor genome carries a derived functional allele (deleterious or adaptive) but is missing from the recipient genome. Thus, M_{add} quantifies the amount of new functional mutations (deleterious or adaptive) that a donor genome can theoretically add in the next generation when crossed with recipient genome.

To measure M_{add}, we first compared, at a given locus l, the genotype of both the donor $\mathbf{g}_{\mathbf{l}}^{d_i}$ and the recipient genome $\mathbf{g}_{\mathbf{l}}^{r_j}$, where d_i = a genome from a donor population and r_j = a genome from a recipient population and g_l = (0|0, 0|1, 1|1). If the functional allele (either deleterious or adaptive) is missing from the recipient genome, i.e., $\mathbf{g}_{\mathbf{l}}^{r_j}$= 0|0 but is present in the donor genome, i.e., $\mathbf{g}_{\mathbf{l}}^{d_i} \neq$ 0|0 then, we count that locus as a mutation added (i.e., $\boldsymbol{m_a^l(d_i, r_j) = 1}$). IN other words, we compared the genotypes of all functional loci between each pair of genomes from donor and recipient populations and counted the loci where the functional allele was present in donor but missing in recipient genome. Thus, for a pair of donor genome d_i and recipient genome r_j, $\boldsymbol{M_{add}(d_i, r_j)}$ is the total number of functional loci where $\boldsymbol{g_l^{di}} \neq \mathbf{0|0}$ & $\boldsymbol{g_l^{rj} = \mathbf{0|0}}$. To get

population level estimates of M_{add}, we averaged the pairwise estimate between each pair of genomes from a donor and a recipient population.

Mutations masked (M_{mask}). Conceptually, M_{mask} measures the number of loci where the recipient genome carries the deleterious allele in homozygous state (i.e., deleterious allele is exposed) but will be masked in heterozygote in the next generation when crossed with the donor genome. Thus, M_{mask} quantifies the number of exposed loci in recipient genome that a donor genome can theoretically mask in the next generation when crossed with recipient genome.

To measure M_{mask}, we first compared, at a given locus l, the genotype of both the donor $\mathbf{g}_{\mathbf{l}}^{d_i}$ and the recipient genome $\mathbf{g}_{\mathbf{l}}^{r_j}$, where d_i = genome from a donor population and r_j = genome from a recipient population and g_l = (0|0, 0|1, 1|1). If the deleterious allele is exposed in the recipient genome in homozygous state, i.e., $\mathbf{g}_{\mathbf{l}}^{r_j}$ = 1|1 but is absent in the donor genome, i.e., $\mathbf{g}_{\mathbf{l}}^{d_i}$ = 0|0 then, we count that locus as a mutation masked $\boldsymbol{m_m^l(d_i, r_j) = 1}$. However, if the donor genome is heterozygous, $\mathbf{g}_{\mathbf{l}}^{d_i}$ = 0|1 then, under the assumption of Mendelian inheritance (i.e. independent assortment of alleles in gametes), the probability of masking of that given locus in the recipient population in the next generation is only 0.5.

Hence, we can divide the M_{mask} into two subcategories $\boldsymbol{M_{mask}^{P=1}}$ and $\boldsymbol{M_{mask}^{P=0.5}}$ depending on the donor genotype, where $\boldsymbol{M_{mask}^{P=1}}$ for a given donor-recipient genome pair is the total number of deleterious loci where $\mathbf{g}_{\mathbf{l}}^{d_i}$ = 0|0 and $\mathbf{g}_{\mathbf{l}}^{r_j}$= 1|1 and $\boldsymbol{M_{mask}^{P=0.5}}$ is the total number of deleterious loci where $\mathbf{g}_{\mathbf{l}}^{d_i}$ = 0|1 and $\mathbf{g}_{\mathbf{l}}^{r_j}$= 1|1.

Mutations unmasked (M_{unmask}). Conceptually, M_{unmask} measures the number of loci where the deleterious allele is in heterozygous state (i.e., deleterious allele is masked) in the recipient genome but will be unmasked or exposed in homozygous state in the next generation when crossed with the donor genome. Thus, M_{unmask} quantifies the number of masked loci in the recipient genome that a donor genome can theoretically unmask in the next generation when crossed with recipient genome.

To measure M_{unmask}, we first compared, at a given locus l, the genotype of both the donor $\mathbf{g}_{\mathbf{l}}^{d_i}$ and the recipient genome $\mathbf{g}_{\mathbf{l}}^{r_j}$, where d_i = genome from a donor population and r_j = genome from a recipient population and g_l = (0|0, 0|1, 1|1). If the deleterious allele is masked in the recipient genome in heterozygous state, i.e., $\mathbf{g}_{\mathbf{l}}^{r_j}$ = 0|1 but is exposed in the donor genome, i.e.,

$\mathbf{g}_{\mathbf{l}}^{d_i}$ = 1|1 then under the assumption of Mendelian inheritance (i.e. independent assortment of alleles in gametes), the probability of unmasking of that given locus in the recipient genome in the next generation is 0.5, whereas, if $\mathbf{g}_{\mathbf{l}}^{d_i}$ = 0|1, then the probability of unmasking of that given locus is only 0.25.

Hence, we can divide the M_{unmask} into two subcategories $\boldsymbol{M_{unmask}^{P=0.5}}$ and $\boldsymbol{M_{unmask}^{P=0.25}}$ depending on the donor genotype, where $\boldsymbol{M_{unmask}^{P=0.5}}$ for a given donor-recipient genome pair is the total number of deleterious loci where $\mathbf{g}_{\mathbf{l}}^{d_i}$ = 1|1 and $\mathbf{g}_{\mathbf{l}}^{r_j}$= 0|1 and $\boldsymbol{M_{unmask}^{P=0.25}}$ is the total number of deleterious loci where $\mathbf{g}_{\mathbf{l}}^{d_i}$ = 0|1 and $\mathbf{g}_{\mathbf{l}}^{r_j}$= 0|.

The overall masking potential (P_{mask}) assumes the Mendelian law of independent assortment i.e., *AA*, *Aa*, and *aa* genotypes, where *A* and *a* are wildtype and deleterious allele, respectively, would segregate independently and in equal proportions in the germline for all loci. These probabilities we assume for masking as estimated from this assumption. Regarding dominance coefficient (h), we assume that no deleterious allele is completely dominant (heterozygote has same fitness as mutant homozygote) and thus, $h < 1$ for all deleterious alleles analyzed in this study. The relative fitness (w) of different genotypes can be defined as:

- $w_{AA} = 1$ (homozygous wild-type)
- $w_{Aa} = 1 - hs$ (heterozygote)
- $w_{aa} = 1 - s$ (homozygous mutant)

where s = selection coefficient.

For masking potential (P_{mask}), we are assuming that deleterious allele would have a negative fitness impact ($s \neq 0$) and the heterozygote fitness would be higher than homozygous mutant genotype (i.e., $w_{Aa} > w_{aa}$). Even if the deleterious allele is completely recessive ($h = 0$), that would just mean that heterozygote is as fit as homozygous wildtype ($w = 1$), both of which are more fit than homozygous mutant genotype ($w = 1$-s).

In conclusion, with the introduction of these metrics, we assume that masking of deleterious allele in heterozygotes would result in higher fitness as compared to exposed deleterious alleles in homozygous mutant genotypes, and vice-versa (i.e., $s \neq 0$ and $h < 1$). For more theoretical explanation see García-Dorado (2012), Bertorelle et al. (2022), and references therein.

References

Bertorelle, G., Raffini, F., Bosse, M., Bortoluzzi, C., Iannucci, A., Trucchi, E., . . . van Oosterhout, C. (2022). Genetic load: genomic estimates and applications in non-model animals. *Nature Reviews Genetics, 23*(8), 492-503. doi:10.1038/s41576-022-00448-x

García-Dorado, A. (2012). Understanding and Predicting the Fitness Decline of Shrunk Populations: Inbreeding, Purging, Mutation, and Standard Selection. *Genetics, 190*(4), 1461-1476. doi:10.1534/genetics.111.135541

Latrille, T., Rodrigue, N., & Lartillot, N. (2023). Genes and sites under adaptation at the phylogenetic scale also exhibit adaptation at the population-genetic scale. *Proceedings of the National Academy of Sciences, 120*(11). doi:10.1073/pnas.2214977120

Mathur, S., Mason, A. J., Bradburd, G. S., & Gibbs, H. L. (2023). Functional genomic diversity is correlated with neutral genomic diversity in populations of an endangered rattlesnake. *Proceedings of the National Academy of Sciences, 120*(43). doi:10.1073/pnas.2303043120

Stoletzki, N., & Eyre-Walker, A. (2010). Estimation of the Neutrality Index. *Molecular Biology and Evolution, 28*(1), 63-70. doi:10.1093/molbev/msq249

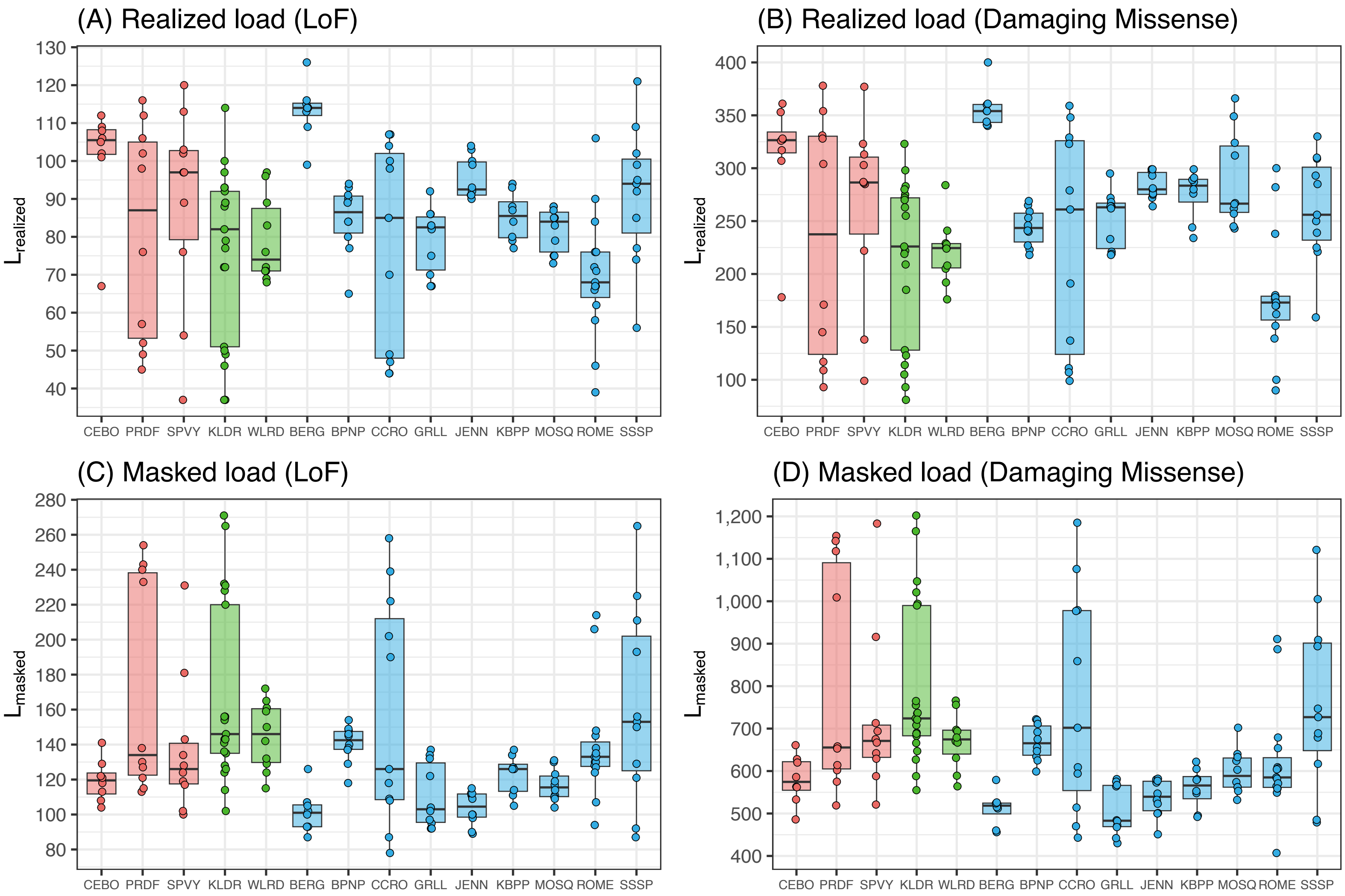

Fig. S1: Mutation load (masked and realized) in different *S. catenatus* populations. Realized load ($L_{realized}$) was measured as the number of SNPs within an individual that are homozygous for the (A) Loss of Function (LoF) mutation and (B) missense mutations labelled as "damaging" by PROVEAN. Masked load (L_{masked}) was measured as the number of SNPs within an individual that are heterozygous at the (C) Loss of Function (LoF) loci and (D) damaging missense mutations. Error bars show 1 $\pm$ SD. Red = Recipients; Green = Donors; Blue = Others.

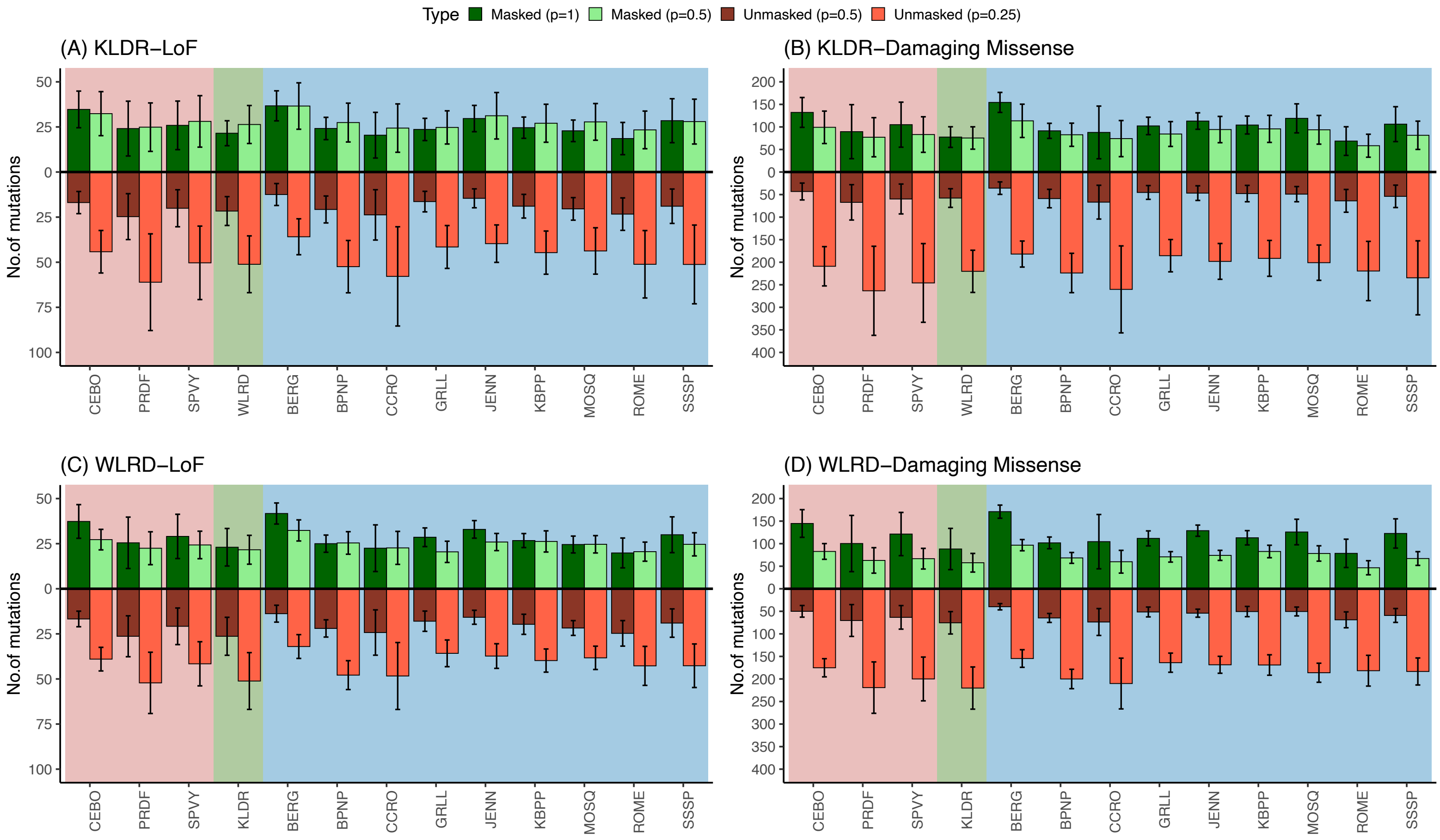

Fig. S2: **Number of mutations masked and unmasked by (A-B) KLDR and (C-D) WLRD donor populations**. Number of exposed (A) LoF or (B) deleterious mutations in different recipient populations that can be masked by KLDR population at probability of 1 (dark green) and 0.5 (light green) in the next generation, and the number of masked LoF or deleterious mutations in different recipient populations that can be exposed by KLDR population at probability of 0.5 (dark red) and 0.25 (light red) in the next generation. (C-D) Similar analysis was performed with WLRD donor population. Bar plots indicate the mean number of mutations per donor-recipient genome pair and error bars represent 1SD. Red box = Recipients, Green box = Donors; Blue Box = Other populations.

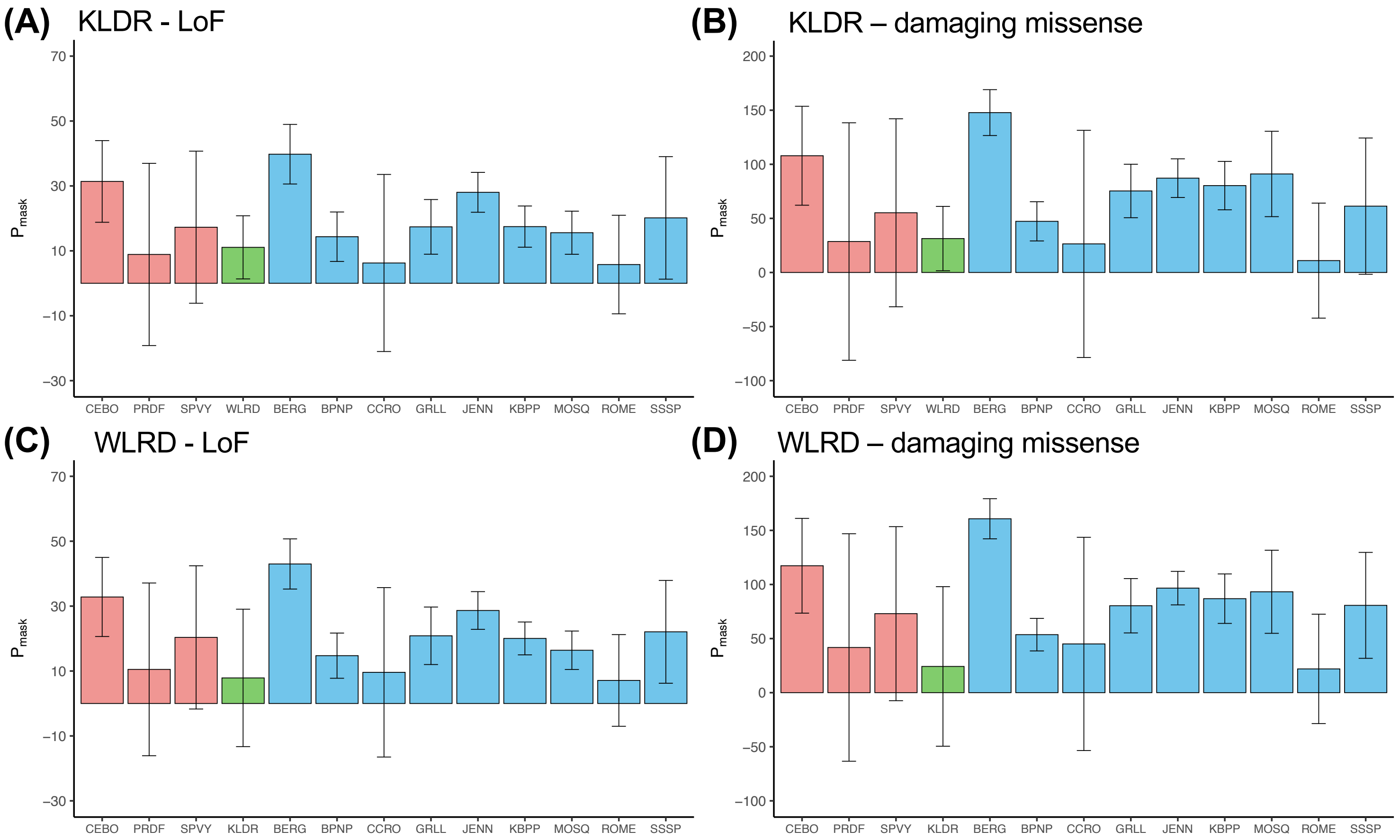

Figure S3: Genetic compatibility assessment based on net masking potential of donor populations to mask target recipient (and other) populations. Panels show the mean masking potential (P_{mask}) score measuring the effective (A) LoF and (B) deleterious mutations masked in heterozygous state in F1 generation of potential recipient (red) and other (blue) populations when crossed with KLDR genomes (see Supplementary methods; Fig. S1). Similar analysis was performed using WLRD genomes as donors (C and D). A positive P_{mask} indicates more chances of masking exposed deleterious mutations whereas a negative P_{mask} indicates a higher chance of exposing masked deleterious mutations. Error bars represent 1 $\pm$ SD

Table S1: No. of samples re-sequenced from each site. Sequencing details for each individual can be found in Data S1.

Pop	No. of samples	State	Label
CEBO	8	OH	Recipient1
PRDF	10	OH	Recipient2
SPVY	10	OH	Recipient3
KLDR	21	OH	Donor1
WLRD	10	OH	Donor2
BERG	8	NY	Other
BPNP	10	CA	Other
CCRO	11	NY	Other
GRLL	10	OH	Other
JENN	10	PA	Other
KBPP	8	CA	Other
MOSQ	10	OH	Other
ROME	15	OH	Other
SSSP	11	IL	Other

Table S2: Mean individual masked (LoadM) and realized load (LoadR) of LoF and damaging missense mutations, and mean adaptive variation per population. Mean +/- 1SD.

Pop	LoF mutations		Damaging Missense mutations		Adaptive diversity
	LoadM	LoadR	LoadM	LoadR	
CEBO	119.5 +/- 11.82	101.25 +/- 14.3	579.88 +/- 56.26	312.12 +/- 57.03	3.150e-04 +/- 1.563e-04
PRDF	171.4 +/- 61.81	81.3 +/- 28.48	804.4 +/- 265.01	233 +/- 115.16	4.310e-04 +/- 1.879e-04
SPVY	137.9 +/- 39.97	88.8 +/- 26.09	722.8 +/- 191.39	263.3 +/- 85.95	4.034e-04 +/- 1.673e-04
KLDR	166.95 +/- 51.07	76 +/- 22.36	795.67 +/- 190.32	214.1 +/- 76.39	4.241e-04 +/- 2.006e-04
WLRD	144.9 +/- 19.38	79.2 +/- 11.23	671.3 +/- 64.38	221.2 +/- 29.43	3.227e-04 +/- 1.638e-04
BERG	101.62 +/- 11.95	113.25 +/- 7.52	511.75 +/- 39.24	356.75 +/- 19.29	2.719e-04 +/- 2.162e-04
BPNP	140.4 +/- 10.64	84.7 +/- 8.9	668.1 +/- 42.75	244.1 +/- 17.66	3.802e-04 +/- 1.500e-04
CCRO	157.91 +/- 65.26	77.73 +/- 27.33	764.36 +/- 261.52	231.27 +/- 104.39	3.898e-04 +/- 1.443e-04
GRLL	110.7 +/- 18.47	79.1 +/- 8.77	506.5 +/- 58.69	251.6 +/- 26.79	2.352e-04 +/- 1.546e-04
JENN	103.6 +/- 9.49	95.4 +/- 5.48	536.6 +/- 43.65	283.5 +/- 12.56	2.018e-04 +/- 1.379e-04
KBPP	122.5 +/- 11.33	85.25 +/- 6.36	559.38 +/- 47.37	275 +/- 23.42	3.201e-04 +/- 1.575e-04
MOSQ	116.8 +/- 9.02	81.8 +/- 5.81	598.5 +/- 51.7	289.1 +/- 44.85	2.483e-04 +/- 1.678e-04
ROME	139.47 +/- 31.64	69.87 +/- 16.37	621.73 +/- 127.39	178.87 +/- 57.2	3.392e-04 +/- 1.810e-04
SSSP	162 +/- 56.12	91.27 +/- 17.83	759.27 +/- 204.55	261.55 +/- 49.99	4.761e-04 +/- 2.115e-04

Table S3: Mean number of functional mutations (LoF, damaging missense, and adaptive) introduced by the two donor populations (KLDR, WLRD) into the recipient populations (CEBO, PRDF, SPVY)

Recipient	Novel LoF mutations added by donor		Novel Damaging Missense mutations added by donor		Novel Adaptive mutations added by donor	
	**KLDR**	**WLRD**	**KLDR**	**WLRD**	**KLDR**	**WLRD**
CEBO	115.3 +/-24.02	104.41 +/-8.71	577.6+/-104.97	499.91+/-40.09	145.77+/-28.08	123.86+/-11.98
PRDF	99.94 +/-26.86	89.77 +/-14.16	535.63+/-116.06	470.24+/-57.86	138.59+/-37.06	120.28+/-24.91
SPVY	109.6 +/-24.98	101.91 +/-10.38	545.75+/-112.27	487.18+/-49.8	132.21+/-31.78	114.74+/-17.88

Table S4: Mean proportion of functional mutations (LoF, damaging missense, and adaptive) introduced by the two donor populations (KLDR, WLRD) into the recipient populations (CEBO, PRDF, SPVY)

Recipient	% Novel LoF mutations added by donor		% Novel Damaging Missense mutations added by donor		% Novel Adaptive mutations added by donor	
	KLDR	WLRD	KLDR	WLRD	KLDR	WLRD
CEBO	34.02+/-1.62	32.09+/-1.71	39.03+/-1.52	35.9+/-1.63	36.24+/-2.33	32.79+/-2.49
PRDF	28.38+/-6.07	26.51+/-5.42	34.15+/-6.02	31.54+/-5.5	33.11+/-7.82	30.34+/-7.7
SPVY	32.38+/-3.46	31.09+/-3.21	35.51+/-4.31	33.22+/-3.84	31.85+/-5.22	29.09+/-4.78

Table S5: Mean number of deleterious mutations (LoF and damaging missense) that are already exposed in the genomes of recipient populations (CEBO, PRDF, SPVY) and can be masked in the next generation when augmented with the two donor populations (KLDR, WLRD) at probability P=1 or P= 0.5 (See Table S1 for details). Similarly, the mean number of masked mutations in recipient genomes that can be exposed or unmasked by the donor genomes in the next generation at P = 0.5 or P =0.25. Numbers represent mean +/- 1 S.D.

**Pop**	**LoF mutations**								**Damaging Missense mutations**							
	**KLDR**				**WLRD**				**KLDR**				**WLRD**			
	**Masked**		**Unmasked**		**Masked**		**Unmasked**		**Masked**		**Unmasked**		**Masked**		**Unmasked**	
	**P=1**	**P=0.5**	**P=0.5**	**P=0.25**	**P=1**	**P=0.5**	**P=0.5**	**P=0.25**	**P=1**	**P=0.5**	**P=0.5**	**P=0.25**	**P=1**	**P=0.5**	**P=0.5**	**P=0.25**
**CEBO**	34.71 +/- 10.16	32.36 +/- 12.21	16.93 +/- 6.14	44.19 +/- 11.78	37.31 +/- 9.33	27.25 +/- 5.69	16.74 +/- 4.32	39.01 +/- 6.55	132.2 +/- 33.09	99.16 +/- 35.98	43.2 +/- 18.71	209 +/- 43.57	144.7 +/- 30.67	82.75 +/- 17.42	50 +/- 12.91	175.2 +/- 20.13
**PRDF**	24.09 +/- 15.19	24.87 +/- 13.43	24.72 +/- 12.72	61.08 +/- 26.8	25.48 +/- 14.25	22.47 +/- 9.11	26.34 +/- 11.35	52.17 +/- 16.99	89.48 +/- 59.9	77.17 +/- 43.39	67.23 +/- 39.19	263.4 +/- 98.77	100.3 +/- 62.35	62.85 +/- 28.24	70.39 +/- 35.29	219.2 +/- 56.96
**SPVY**	25.87 +/- 13.46	28.07 +/- 14.27	20.09 +/- 10.26	50.34 +/- 20.37	29.02 +/- 12.27	24.29 +/- 7.63	20.81 +/- 10.14	41.6 +/- 12.23	105 +/- 49.94	83.11 +/- 39.41	59.78 +/- 33.14	246 +/- 87.25	121.4 +/- 47.91	66.7 +/- 22.89	63.39 +/- 26.13	200 +/- 48.56

Table S6: Mean proportion of deleterious mutations (LoF and damaging missense) that are already exposed (loadR) in the genomes of recipient populations (CEBO, PRDF, SPVY) and can be masked in the next generation when augmented with either of the two donor populations (KLDR, WLRD) at probability P=1 or P= 0.5 (See Table S1 for details). Similarly, the mean proportion of masked mutations in recipient genomes (LoadM) that can be exposed or unmasked by the donor genomes in the next generation at P = 0.5 or P =0.25. Numbers represent mean percentage +/- 1 S.D.

Pop	LoF mutations								Damaging Missense mutations							
	KLDR				WLRD				KLDR				WLRD			
	% Masked		% Unmasked		% Masked		% Unmasked		% Masked		% Unmasked		% Masked		% Unmasked	
	P=1	P=0.5	P=0.5	P=0.25	P=1	P=0.5	P=0.5	P=0.25	P=1	P=0.5	P=0.5	P=0.25	P=1	P=0.5	P=0.5	P=0.25
**CEBO**	33.68 +/- 5.56	32.05 +/- 1.02	14.11 +/- 1.76	37.02 +/- 1.37	36.21 +/- 6.08	26.98 +/- 1.23	13.98 +/- 1.72	32.71 +/- 2.83	41.94 +/- 2.85	31.86 +/- 1.19	7.38 +/- 1.3	36.07 +/- 1.32	46.04 +/- 2.77	26.56 +/- 0.86	8.57 +/- 1.43	30.22 +/- 0.68
**PRDF**	26.59 +/- 9.78	30.27 +/- 1.77	14.31 +/- 1.49	35.98 +/- 1.84	28.67 +/- 8.68	27.47 +/- 1.81	15.16 +/- 1.68	31.06 +/- 2.47	33.92 +/- 10.61	33.53 +/- 1.65	8.05 +/- 1.49	33.03 +/- 2.02	38.91 +/- 10.49	27.77 +/- 2.57	8.31 +/- 1.99	27.87 +/- 2.41
**SPVY**	27.21 +/- 7.5	31.3 +/- 1.66	14.34 +/- 2.4	36.6 +/- 2.31	30.85 +/- 7.25	27.73 +/- 1.99	14.72 +/- 3.05	30.38 +/- 1.87	37.59 +/- 8.47	31.63 +/- 1.3	8.11 +/- 1.44	34.19 +/- 2.73	44.04 +/- 7.52	25.24 +/- 1.34	8.59 +/- 1.5	27.95 +/- 2.42

Table S7: Mean masking potential of donor population (KLDR, WLRD) to mask deleterious mutations (LoF and damaging missense) in the genomes of recipient populations (CEBO, PRDF, SPVY). Numbers represent mean +/- 1 S.D.

Recipient Population	LoF mutations		Damaging Missense mutations	
	KLDR	WLRD	KLDR	WLRD
CEBO	31.38+/-12.58	32.82+/-12.18	107.89+/-45.73	117.31+/-43.79
PRDF	8.89+/-28.07	10.5+/-26.61	28.61+/-109.75	41.75+/-105.13
SPVY	17.28+/-23.43	20.36+/-22.07	55.2+/-86.89	73.02+/-80.43

Supplementary Data

Data S1. Table containing list of all *S. catenatus* whole genome sequences analyzed in this study (N=152). Columns include sample location, State, number of paired-end sequence reads for each sample, mean depth of coverage, mean breadth of coverage of each sample at 1x and 10x depth, the population label (Donor, Recipient, Other), and SRR number of previously sequenced individuals. (**DataS1.xlsx**)
